# Molecular Subtype Conversion between Primary and Metastatic Breast Cancer Corresponding to the Dynamics of Apoptotic and Intact Circulating Tumor Cells

**DOI:** 10.3390/cancers11030342

**Published:** 2019-03-11

**Authors:** Stefan Stefanovic, Thomas M. Deutsch, Ralph Wirtz, Andreas Hartkopf, Peter Sinn, Florian Schuetz, Christof Sohn, Michael K. Bohlmann, Marc Sütterlin, Andreas Schneeweiss, Markus Wallwiener

**Affiliations:** 1 Department of Gynecology and Obstetrics, Mannheim University Hospital, University of Heidelberg, Theodor-Kutzer-Ufer 1-3, 68167 Mannheim, Germany; michael.bohlmann@umm.de (M.K.B.); Marc.Suetterlin@umm.de (M.S.); 2 Department of Gynecology and Obstetrics, Heidelberg University Hospital, Im Neuenheimer Feld 440, 69120 Heidelberg, Germany; thomas.deutsch@med.uni-heidelberg.de (T.M.D.); Florian.Schuetz@med.uni-heidelberg.de (F.S.); christof.sohn@med.uni-heidelberg.de (C.S.); andreas.schneeweiss@med.uni-heidelberg.de (A.S.); markus.wallwiener@med.uni-heidelberg.de (M.W.); 3 Stratifyer Molecular Pathology GmbH, Werthmannstr. 1c, 50935 Cologne, Germany; ralph.wirtz@stratifyer.de; 4 Department of Women’s Health, University Hospital Tübingen, Calwerstr. 7, 72076 Tübingen, Germany; andreas.hartkopf@med.uni-tuebingen.de; 5 Department of Pathology, Heidelberg University Hospital, Im Neuenheimer Feld 224, 69120 Heidelberg, Germany; peter.sinn@med.uni-heidelberg.de; 6 National Center for Tumor Diseases (NCT) Heidelberg, Im Neuenheimer Feld 460, 69120 Heidelberg, Germany

**Keywords:** breast cancer, intrinsic subtype, biomarker conversion, circulating tumor cells, RT-qPCR

## Abstract

The presence of circulating tumor cells (CTCs), detected as a form of liquid biopsy is associated with poor survival in both early and metastatic breast cancer. Monitoring tumor biology based on intrinsic subtypes delivers treatment-relevant information on the heterogeneity or biomarker conversion between primary and metastatic tumors. This study aimed to correlate the change of the apoptotic and intact CTC counts with mRNA-assessed intrinsic subtype change. Thirty-four breast cancer patients with available triplets of primary tumors, distant metastasis biopsies and data on intact and apoptotic CTC dynamics were included in the analysis. The intrinsic subtype was determined per RT-qPCR quantification of the gene expression ESR1, PGR, ERBB2 and MKI67. Both luminal (*p* = 0.038) and triple negative (*p* = 0.035) patients showed a significant downregulation of apoptotic CTCs. Repeated biopsies of distant metastatic sites, as well as determining a potential shift of the intrinsic subtype, combined with data on intact and apoptotic CTC dynamics from liquid biopsies might help personalize systemic therapy and generate additional surrogate markers for successful systemic therapy.

## 1. Introduction

Performing a liquid biopsy by repeatedly measuring the rates of circulating tumor cells (CTCs) or circulating tumor DNA (ctDNA) rates in serum is a non-invasive method of monitoring disease activity in all phases of breast cancer [1,2,3,4]. CTCs have been confirmed as prognosticators of poor survival in both early and metastatic breast cancer and comprise a subset of morphologically altered breast cancer cells with metastasis-initiating capabilities [5,6].

Determining the tumor biology, preferably based on the intrinsic subtype, delivers additional information on tumor heterogeneity or tumor marker conversion between primary (PT) and metastatic tumors (MT) [7,8]. 

We have previously reported intrinsic subtype-related and site-related discrepancies in tumor biology between primary and metastatic tumors by mRNA-assessment, possibly based on tumor heterogeneity or biomarker conversion, underlining the importance of re-biopsies and biopsies of distant metastases. Simultaneously, a high level of concordance between the RT-qPCR and protein analysis was observed [9]. 

Conventional re-biopsies and biopsies of distant metastatic sites are often not feasible or are highly invasive. Consequently, alternative methods for following disease activity and analyzing tumor biomarker dynamics, including liquid biopsies, were devised. Since enumerating CTCs requires only a few milliliters of the whole blood, determining the CTC count is particularly useful in a practical context. Aside from the possibility of tumor biology monitoring per se, the detection of CTCs also has a prognostic value [10]. The presence of at least five CTCs in 7.5 mL of blood is associated with reduced progress-free survival (PFS) and overall survival (OS) in patients with metastatic breast cancer (MBC) [11]. Thus far, several studies have demonstrated the usefulness of serial CTC enumeration as a means of monitoring the efficacy of therapy [12].

The results of the prospective phase III STIC CTC trial imply that CTC rates might represent a criterion when deciding on first line endocrine treatment versus chemotherapy in occurrences of luminal metastatic breast cancer. However, during the follow-up study, the CDK4/6 inhibitors, combined with endocrine therapy, emerged as a favorable first line therapy, thus limiting the impact of the STIC CTC conclusions [13].

Apart from their tumorigenic potential and stem-like characteristics, the CTCs are not a monolithic cell population, but a conglomerate of viable and apoptotic/malformed CTCs. Apoptotic CTCs (aCTC), which have been reported in 52–79% of CTC-positive MBC patients, are characterized by altered morphological parameters such as a speckled pattern of keratin staining and fragmented or disintegrated nuclei [1,3,5,12,14,15,16,17]. Most CTCs are cleared from circulation within a few days, especially in the setting of adequate systemic therapy. We were able to demonstrate that both intact and apoptotic CTCs can predict outcomes in metastatic breast cancer and should therefore be enumerated separately. The apoptotic CTC fraction was recognized as associated with a poor prognosis [14].

The present study aimed to assess changes in apoptotic, intact and overall rates of circulating tumor cells after one cycle of systemic therapy, according to the intrinsic subtype, and to correlate the change of the CTC count with mRNA-measured biomarker conversion. 

## 2. Results

Thirty-four breast cancer patients from the National Center for Tumor Diseases (NCT, Heidelberg, Germany) CONCORD database, with available triplets of biopsies of the primary tumor (PT), a biopsy of a metastatic tumor site (MT), as well as data on CTC dynamics, were included in the analysis (Table 1).

Hereby, being measured by RT-qPCR, 19 (55.9%) patients converted their intrinsic subtype, compared to 14 (41.2%) stable ones (Table 1). The intrinsic subtype shifted towards a triple negative phenotype with 10 (29.4%) triple negative patients at MT versus 7 (20.6%) at PT. A drop in the luminal patient count has been also observed (Table 1).

The population of CTC-positive patients crucially decreased between the baseline liquid biopsy and the follow-up measurement after one cycle of systemic cytotoxic therapy (in the case of triple negative patients), i.e., after 3 cycles of endocrine therapy (in the case of luminal patients), in terms of the whole-CTC count of 23 (67.6%) versus 11 (32.4%), as well as aCTC count of 16 (47.1%) versus 7 (20.6%) and an intact CTC (iCTC) count of 23 (67.6%) versus 11 (32.4%) (Table 1).

Additionally, the RT-qPCR data have proven to be highly concordant with the immunohistochemistry-based tumor subtype assessment (Table 1).

**Figure 1:**
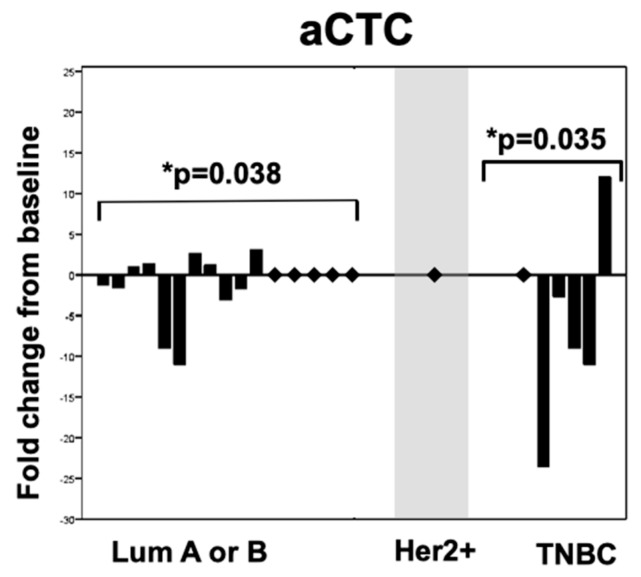
Changes in rates of apoptotic (aCTC) circulating tumor cells after one cycle of systemic therapy according to the intrinsic subtype of the metastatic tumor.

Based on the mRNA-assessed biopsy of the metastatic tumors, upon one cycle of systemic therapy, both luminal (*p* = 0.038) and triple negative (*p* = 0.035) patients show a significant downregulation of apoptotic CTCs (Figure 1). 

**Figure 2:**
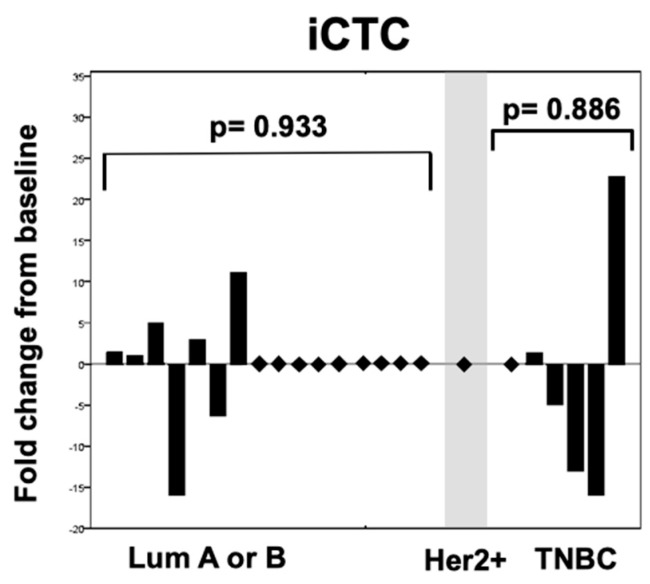
Changes in rates of intact (iCTC) circulating tumor cells after one cycle of systemic therapy according to the intrinsic subtype of the metastatic tumor.

**Figure 3:**
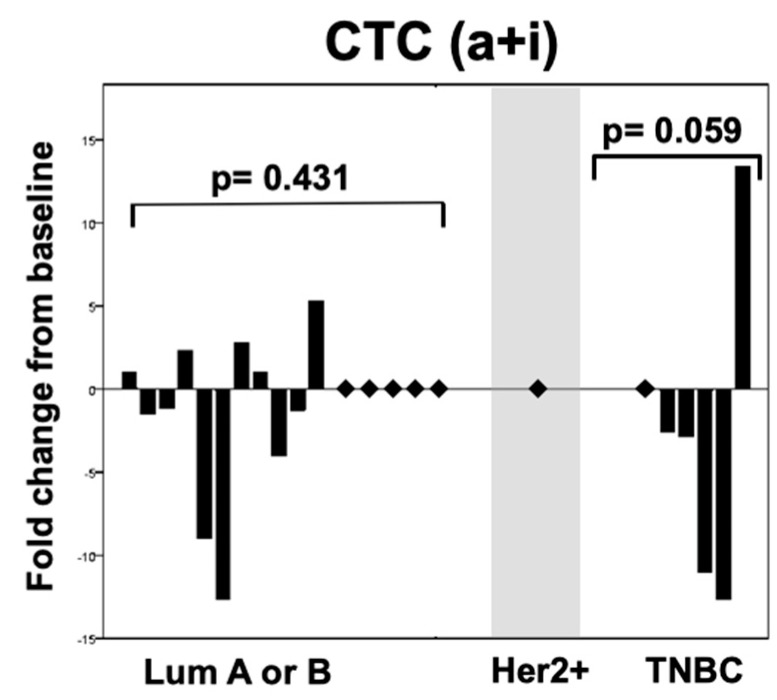
Changes in rates of overall (a+iCTC) circulating tumor cells after one cycle of systemic therapy according to the intrinsic subtype of the metastatic tumor.

The observed significant depletion of apoptotic CTCs stands in contrast when compared to an insignificant change of intact CTCs (*p* = 0.933 for luminal and *p* = 0.886 for triple negative patients), as well as the whole-CTC population in all subtypes (*p* = 0.431 and 0.059 for luminal and triple negative patients respectively) (Figure 2 and Figure 3).

Further subgroup analyses were performed according to the modality of systemic therapy (Table 2). A vast majority of the patients received chemotherapy (70.6%), followed by endocrine therapy (26.5%) and anti-HER2 therapy (2.9%). The respectively high rates of CTC positivity expressed prior to chemotherapy begin, then rapidly decline after one cycle of cytotoxic treatment, decreasing from 58.8% to 29.4% for intact CTCs, which is even more evident for the apoptotic CTC fraction, which reduces from 41.2% to 17.6% after one cycle of chemotherapy. The initially lower rates of CTCs in patients under endocrine treatment showed an overall decline from 8.8% to 2.9% for intact CTCs and from 5.9% to 2.9% for apoptotic CTCs (Table 2).

## 3. Discussion

Analyzing the changes of tumor biomarkers and monitoring treatment response are of vast importance for the prognosis and further personalized treatment decision-making in incidences of metastatic breast cancer [18,19,20]. 

Circulating tumor cells have been demonstrated to be a prognostic factor in both early and metastatic breast cancer settings. The follow-up data from the Success A study (an open-label multicenter phase III study with high-risk early breast cancer patients, in which the patients with high-risk primary breast cancer were first randomized to be given three cycles of epirubicin-fluorouracil-cyclophosphamide, followed by either three cycles of docetaxel or three cycles of gemcitabine-docetaxel, followed by a second randomization of either two or five years of zoledronate treatment) emphasize that CTCs five years after chemotherapy are associated with decreased recurrence-free survival, suggesting CTC persistence within a long-term follow-up as an independent predictor of late recurrences in hormone receptor-positive patients [21].

Our data, showing both intact and apoptotic positivity, predominantly in the aggressive intrinsic subtype/higher therapy-pressure setting (20 CTC positive out of 24 under chemotherapy at baseline versus 3 out of 9 undergoing endocrine treatment), are in line with the aforementioned findings.

Furthermore, in the metastatic setting, a pooled analysis from Bidard et al. on *n* = 1.944 patients has confirmed a favorable PFS and OS prognosis for CTC-negative breast cancer patients [11,22].

Data from the DETECT study family, which comprises a major study cluster evaluating therapeutic intervention based on the characterization of CTC expression phenotypes in patients with metastatic breast cancer, are expected to elaborate the predictive value of CTCs and shed new light on treatment response monitoring by evaluating CTC expression profiles [23,24].

### 3.1. Role of Apoptotic CTCs as a Potential Parameter of Treatment Response

Our study was able to confirm a trend in biomarker conversion towards a more aggressive intrinsic subtype in the course of breast cancer metastatic progression, with an increase of triple negative patients and a decrease of luminal biopsies in distant metastases (Table 1).

In particular, having the levels of the apoptotic CTC fraction significantly drop in both the luminal (*p* = 0.038) and triple negative subpopulations (*p* = 0.035) (Figure 1) underlines the importance of differentiating between iCTCs and aCTCs within CTC enumeration, speaking in favor of the aCTC fraction as a parameter of treatment response in metastatic breast cancer. 

This aspect of the observation is particularly in line with the findings of our previous study [14]. There, the determination of the kinetics of apoptotic CTCs showed stronger discriminating power than the kinetics of the intact CTCs, with higher PFS and OS for the group with decreasing CTCs levels. Different types of systemic treatment had no independent influence on the aCTCs [14].

### 3.2. Therapy Modalities and Possible Implications for CTC Dynamics

Apoptotic CTCs can possibly emerge within therapy-induced apoptosis and/or from spontaneous tumor apoptosis, since they are detected in patients with progressive disease and a lack of response to systemic therapy [14,25,26].

It is suggested that the viability of CTCs is related to the stage of disease and, aCTCs might provide additional prognostic information, mostly speaking in favor of the hypothesis that aCTCs might serve as a surrogate marker of successful systemic therapy [25,26,27,28]. 

It should be emphasized that due to the spontaneous depletion of aCTCs in the course of the metastatic disease, independent from treatment response, aCTCs are also to some extent regarded as a side product of metastatic tumors and therefore should be interpreted only as an extended surrogate marker of treatment response in metastatic settings [14,29].

The remaining intact CTCs, being present in 29.4% of the patients that have undergone one cycle of chemotherapy, might be involved in disease progression [27].

### 3.3. Limitations

A major limitation of our study, leading to low power and partially non-significant *p*-values, is the rather small patient cohort. The fairly small patient collective is a result of demanding inclusion criteria requiring available triplets of primary tumors and metastatic tumor biopsies, both analyzed by RT-qPCR for the intrinsic subtype, as well as liquid biopsy data, differentiating between viable and apoptotic CTCs at a baseline and after one cycle of systemic treatment. Taking the aforementioned information into consideration, our findings are foremost hypothesis-generating. One of the interesting topics we are keen to address in future is the correlation of HER2 expression on CTCs with brain metastases, which was not possible in this study due to a lack of brain metastasized patients among those meeting the inclusion criteria (available triplets). On the other hand, numerous patients from our CTC database with cerebral metastases lack mRNA data on biomarker conversion and were therefore not eligible for inclusion. Another technical limitation was caused by the inability of the CellSearch^TM^ system to detect epithelial cell adhesion molecule (EpCAM)/keratin-negative CTCs, which compromises the sensitivity of our tool. EpCAM-based systems in particular may miss cells in the process of epithelial-mesenchymal transition (EMT) [15], which in turn results in underestimation of CTC rates [14].

## 4. Materials and Methods

### 4.1. Patients

The analysis included patients with metastatic breast cancer (MBC) that were treated at the Heidelberg University Hospital, Germany, from April 2011 through to May 2015. Patients with any TNM stage at presentation and any metastatic entity were considered for the study. In order to be included in the study, the patients had to have undergone both primary (PT) and metastatic tumor (MT) biopsies and had to have had blood samples collected within 12 months since metastasis detection. The tissue samples acquired by biopsy were all formalin-fixed and paraffin-embedded (FFPE) and stored with blood samples as matched triplets provided by the tissue bank of the National Center for Tumor Diseases (NCT, Heidelberg, Germany) in accordance with the regulations of the tissue bank and the approval of the ethics committee of the Medical Faculty of the University of Heidelberg, approval no. S-295/2009. Thirty-four such triplets were analyzed in the study. Demographic data and clinical characteristics were described as frequencies and percentages, means and standard deviations. Immunohistochemistry was conducted according to international guidelines [30].

### 4.2. Real Time qPCR (RT-qPCR) and Defining Intrinsic Subtypes

Each tissue sample (both from the PT and MT) was examined by a pathologist in order to establish that it indeed contained cancer. Subsequently, a single whole-face 10 μm-thick section of each tumor block was processed with a commercial RNA extraction kit (RNXtract^®^, BioNTech Diagnostics GmbH, Mainz, Germany). Having extracted the RNA, a commercial RT-qPCR kit (MammaTyper^®^, BioNTech Diagnostics GmbH, Mainz, Germany) was utilized in order to quantify the relative gene expression of ESR1, PGR, ERBB2 and MKI67 and two other reference genes (B2M and CALM2), in accordance with a pre-established protocol. After a single cycle of primer-specific reverse transcription, followed by 40 cycles of amplification, a ΔΔCq value was calculated for each of the four aforementioned genes of interest (GOI) by using a well-established mathematical model [31,32].

Upon dichotomizing continuous qRNA values, the molecular subtype of each tumor was defined [31,33]. Luminal A high *ESR1* or *PGR* mRNA content and a low *ERBB2* and *MKI67* content was considered characteristic of the luminal A phenotype, a high cancer and *MKI67* content, or a high *ESR1* content but a low *PGR* and *ERBB2* content of the Luminal B phenotype, while triple-negative cancers had to be associated with a low *ESR1, PGR* and *ERBB2* mRNA content. Cut-offs for the markers ERBB2, ESR1 and PGR were defined based on our previous publication, according to an independent technical cohort [31].

### 4.3. Quantification of Circulating Tumor Cells

Total circulating tumor cell enumeration (CTC), as well as intact (iCTC) and apoptotic circulating tumor cell enumeration (aCTC) were performed using CellSearch™. All analyses were evaluated at baseline and after the first cycle of systemic therapy, i.e., three cycles of antihormonal therapy, respectively (iCTC_BL_, aCTC_BL_, iCTC_1C_, and aCTC_1C_), and considered results positive if five or more CTC per 7.5 mL of blood were detected. The CellSearch™ assay (CellSearch™ Epithelial Cell Kit/CellSpotter™ Analyzer, Janssen Diagnostics, LLC, Raritan, NJ, USA) uses a ferrofluid coated with antibodies against epithelial cell adhesion molecule (EpCAM) to separate epithelial cells from peripheral whole blood samples [29,34,35]. Cells separated in such a manner were subsequently immunostained with monoclonal antibodies specific for keratins and CD45. CTC detection was performed by trained observers using a semi-automated fluorescence-based microscopy system (CellSpotter™ Analyzer) [36,37]. Morphologically intact, CD45-negative CTCs without obvious alterations of nuclei and non-speckled keratin immunofluorescence were defined as iCTCs and enumerated by trained operators. Patients with iCTC counts of 5 or higher in any given 7.5 mL blood sample were considered iCTC-positive.

The aCTCs were further characterized by morphological criteria, including fragmented/ disintegrated nuclei and/or speckled keratin staining patterns. In certain patients, the aCTC status was additionally verified by M30 antibody staining, based on the recognition of caspase-cleaved keratin-18 (VLV bio, 1:100) in the fourth channel of the CellSearch system.

The Fisher exact test/Chi square test was used to estimate the statistical significance of the difference in the number of overall CTCs, aCTCs and iCTCs at baseline between those patients that suffered intrinsic subtype conversion and those that did not. Changes in aCTC, iCTC and overall CTC counts from baseline to levels after one cycle of systemic therapy were determined for each patient. The Mann–Whitney U test was used assess the differences between the molecular subtypes in terms of aCTC, iCTC and the overall CTC dynamics before and after the first therapy cycle.

## 5. Conclusions

The comparison of tumor biology between primary tumors and distant metastatic tumors, based on mRNA analysis regarding stability, i.e., through the conversion of the intrinsic subtype and conversion of particular biomarkers, especially in synergy with data on the dynamics of the apoptotic fraction of circulating tumor cells, could help develop new prognostic surrogate markers and treatment response parameters for breast cancer.

Therefore, consequent biopsies of distant metastatic sites and determining a potential shift of the intrinsic subtype, combined with data on intact and apoptotic CTC acquired from liquid biopsies, might help individualize systemic therapy based on the current tumor biology and could generate additional prognosticators of successful systemic therapy.

## Figures and Tables

**Table 1:** Clinicopathological patient characteristics.

**Patient characteristic**	***n* (%)**	
Total, *n*	34	
Mean age at primary tumor biopsy, years	52.3	
Mean age at metastatic tumor biopsy, years	58.1	
**Phenotype of primary tumor, immunohistochemistry**	***n* (%)**	
Luminal A	18 (53%)	
Luminal B	9 (26.5%)	
HER2 positive	2 (6%)	
Triple-negative	4 (12%)	
NA	1 (2.9%)	
**Intrinsic subtype of primary tumor by RT-qPCR**	***n* (%)**	
Luminal A	16 (47.1%)	
Luminal B	9 (26.5%)	
HER2 positive	1 (2.9%)	
Triple-negative	7 (20.6%)	
NA	1 (2.9%)	
**Intrinsic subtype of metastatic tumor by RT-qPCR**	***n* (%)**	
Luminal A	11 (32.4%)	
Luminal B	11 (32.4%)	
HER2 positive	1 (2.9%)	
Triple-negative	10 (29.4%)	
NA	1 (2.9%)	
**Intrinsic subtype conversion assessed by RT-qPCR**	***n* (%)**	
Converters	19 (55.9%)	
Stable	14 (41.2%)	
NA	1 (2.9%)	
**Grading of primary tumor**	***n* (%)**	
G1	0 (0%)	
G2	18 (53%)	
G3	12 (35%)	
GX	4 (12%)	
**ER/*ESR1* status of primary tumor**	**IHC, *n* (%)**	**RT-qPCR, *n* (%)**
Positive	24 (70.6%)	26 (76.5%)
Negative	10 (29.4%)	8 (23.5%)
Unknown	0 (0%)	0 (0%)
**PR/*PGR* status of primary tumor**	**IHC, *n* (%)**	**RT-qPCR, *n* (%)**
Positive	26 (76.5%)	17 (50%)
Negative	8 (23.5%)	17 (50%)
Unknown	0 (0%)	0 (0%)
**HER2/*ERBB2* status of primary tumor**	**IHC, *n* (%)**	**RT-qPCR, *n* (%)**
Positive	2 (5.9%)	1 (2.9%)
Negative	29 (85.3%)	32 (94.1%)
Unknown	3 (8.8%)	1 (2.9%)
**Ki67/MKI67 status of primary tumor**	**RT-qPCR, *n* (%)**	
Positive	6 (17.6%)	
Negative	27 (8.8%)	
Unknown	1 (2.9%)	
**CTC positive patients at baseline**	***n* (%)**	
CTC_(a+i)_ intact and apoptotic	23 (67.6%)	
CTC_(a)_ apoptotic	16 (47.1%)	
CTC_(i)_ intact	23 (67.6%)	
**CTC positive patients after 1 cycle of systemic therapy**	***n* (%)**	
CTC_(a+i)_	11 (32.4%)	
CTC _(a)_	7 (20.6%)	
CTC _(i)_	11 (32.4%)	

**Table 2:** Therapy modalities and dynamics of CTC rates.

Characteristic	Chemotherapy	Endocrine Therapy	Anti-HER2 Therapy
Total patients, *n (%)*	24 (70.6%)	9 (26.5%)	1 (2.9%)
Age at first diagnosis	51.2	53	46
PFS since study recruitment (months)	7	22	10
OS since study recruitment (months)	16	32	20
**CTC positive patients at baseline, n (%)**			
CTC_(a+i)_ intact and apoptotic	20 (58.8%)	3 (8.8%)	0
CTC_(a)_ apoptotic	14 (41.2%)	2 (5.9%)	0
CTC_(i)_ intact	20 (58.8%)	3 (8.8%)	0
**CTC positive patients after 1 therapy cycle, n (%)**			
CTC_(a+i)_ intact and apoptotic	10 (29.4%)	1 (2.9%)	0
CTC _(a)_ apoptotic	6 (17.6%)	1 (2.9%)	0
CTC _(i)_ intact	10 (29.4%)	1 (2.9%)	0
